# Reticular dysgenesis caused by AK2 deficiency: clinical spectrum and hematopoietic stem cell transplantation outcomes in 10 patients from a single-center

**DOI:** 10.3389/fimmu.2026.1829403

**Published:** 2026-04-23

**Authors:** Bothainah Alaqeel, Faiz Aljohani, Nora Alrumayan, Ali Al-Ahmari, Reem Mohammed, Hawazen Alsaedi, Mouhab Ayas, Sultan Albuhairi, Sahar Elshorbagi, Rand Arnaout, Anas M. Alazami, Bander Alsaud, Hamoud Al-Mousa

**Affiliations:** 1 Section of Pediatric Allergy and Immunology, Department of Pediatrics, King Faisal Specialist Hospital and Research Centre, Riyadh, Saudi Arabia; 2 Section of Stem Cell Transplantation, Department of Pediatric Hematology/Oncology, King Faisal Specialist Hospital and Research Centre, Riyadh, Saudi Arabia; 3 Department of Translational Genomics, Genomic Medicine Centre of Excellence, King Faisal Specialist Hospital & Research Center, Riyadh, Saudi Arabia

**Keywords:** adenylate kinase 2, AK2, hematopoietic stem cell transplant, immunodeficiency, inborn error of immunity, reticular dysgenesis, SCID, severe combined immunodeficiency disease

## Abstract

**Introduction:**

Reticular dysgenesis (RD), caused by biallelic variants in *AK2*, represents the most severe and rare form of Severe Combined Immunodeficiency, characterized by profound defects in lymphoid and myeloid lineages; however, data on its clinical spectrum and hematopoietic stem cell transplantation (HSCT) outcomes remain scarce.

**Methods:**

In this retrospective single-center study, we analyzed genetically confirmed *AK2*-related RD cases managed at King Faisal Specialist Hospital and Research Centre between 2005 and 2025, reviewing clinical, immunologic, genetic, and transplant-related data.

**Results:**

Ten patients from eight unrelated families were included, most with parental consanguinity, all presenting in the neonatal period with severe infections, neutropenia unresponsive to granulocyte colony–stimulating factor, and bilateral sensorineural hearing loss. A recurrent homozygous missense variant (*AK2*: NM_001625.4: c.524G>C; p. Arg175Pro) was identified in nine patients, while one patient harbored a start-loss variant. Seven patients underwent HSCT at a median age of 4 months using matched sibling, haploidentical, or cord blood donors; six survived, yielding a post-transplant survival of 85.7% with a median follow-up of 10 years, and achieved full donor myeloid and lymphoid engraftment with robust immune reconstitution.

**Discussion:**

These findings demonstrate that *AK2*-related RD presents with a distinctive neonatal phenotype and carries high pre-transplant mortality, while early HSCT enables durable engraftment and favorable long-term outcomes, supporting the importance of early diagnosis and newborn screening in high-consanguinity populations.

## 1 Introduction

Severe combined immunodeficiency (SCID) encompasses a heterogeneous group of inherited disorders characterized by profound defects in cellular and humoral immunity, resulting in marked susceptibility to life-threatening infections early in life (1). It also includes a wide range of genetic defects that disrupt lymphocyte development, survival, or function, leading to variable immunophenotypes defined by the presence or absence of T, B, and natural killer (NK) cells (2, 3). Reticular dysgenesis (RD) is the most critical and rarest form of SCID, accounting for <2% of reported cases. Unlike other SCID subtypes, RD is uniquely characterized by combined defects in lymphoid and myeloid lineages, resulting in lymphopenia accompanied by severe neutropenia refractory to granulocyte colony–stimulating factor (G-CSF). Typically, RD clinically presents at birth or in the early neonatal period with aggressive infections, agranulocytosis, and characteristic bilateral sensorineural hearing loss. Bone marrow examination frequently reveals a maturation arrest of myeloid precursors at the promyelocyte stage, highlighting the profound hematopoietic failure that distinguishes RD from other forms of SCID (4).

The molecular basis of RD lies in biallelic pathogenic variants in the *AK2* gene, which encodes adenylate kinase 2, a mitochondrial enzyme ubiquitously expressed in hematopoietic cells. AK2 is located in the mitochondrial intermembrane space, where it catalyzes the reversible transfer of a phosphate group between adenosine triphosphate and adenosine monophosphate to produce adenosine diphosphate, thereby playing a pivotal role in mitochondrial energy homeostasis. Loss of *AK2* function disrupts cellular energy metabolism, resulting in impaired survival, proliferation, and differentiation of hematopoietic progenitors, with particularly grave effects on granulopoiesis and lymphopoiesis. Although erythroid and megakaryocytic lineages are generally preserved, anemia and thrombocytopenia have been observed in some affected patients (5, 6).

HSCT remains the only life-saving and curative therapy for RD. It has the potential to restore immune and hematopoietic functions. However, due to the extreme rarity of this disorder, published data on clinical presentation, transplant strategies, immune reconstitution, and long-term outcomes remain scarce. The largest multicenter cohort to date, encompassing patients from Europe, Asia, and North America, included 32 patients from 29 families and demonstrated substantial heterogeneity in *AK2* gene variants (7). Despite this important contribution, existing experience remains fragmented, highlighting the need for additional well-characterized cohorts to refine management approaches and improve patient outcomes.

This study reports the clinical, immunological, genetic, and HSCT outcomes of 10 patients with *AK2*-related RD treated at a single tertiary care center. It aimed to expand existing knowledge by detailing disease presentation, hematologic and immunologic features, HSCT approaches, engraftment, immune reconstitution, and survival outcomes, thereby providing meaningful real-world data to guide the management of this rare and life-threatening disorder.

## 2 Methods

### 2.1 Patients

This retrospective observational study included 10 patients with molecularly confirmed *AK2* deficiency who were diagnosed and followed up at the Immunodeficiency Clinics of King Faisal Specialist Hospital and Research Centre (KFSHRC) in Riyadh, Saudi Arabia, between January 2005 and December 2025. Their medical records were reviewed to collect detailed information on demographic data, clinical presentation, immunological and genetic findings, HSCT procedures, posttransplant complications, immune reconstitution, and outcomes at the latest available follow-up. The study protocol was reviewed and approved by the Institutional Review Board of KFSHRC (IRB approval number: RAC#2251697; approval date: 23/10/2025). In accordance with institutional and international ethical standards, written informed consent for the publication of any potentially identifiable clinical information or images was obtained from all participants or, for pediatric patients, from their legal guardians.

### 2.2 Cellular and immunological assays

Peripheral blood lymphocyte subsets were quantified via multiparameter flow cytometry following immunofluorescent labeling. Monoclonal antibodies directed against T lymphocytes (CD3, CD4, CD8), B lymphocytes (CD19), and NK cells (CD16 and CD56) were utilized according to the manufacturer’s protocols (Becton, Dickinson & Co., Franklin Lakes, NJ, USA). T-cell functional competence was assessed *in vitro* by measuring lymphocyte proliferative responses after stimulation with phytohemagglutinin (PHA) using established protocols (8). Serum immunoglobulin concentrations, including IgG, IgA, and IgM, were determined via nephelometric analysis in the clinical immunology laboratory.

### 2.3 Genetic analysis

Clinical whole-exome sequencing was performed as part of standard patient care, either outsourced or conducted in-house at College of American Pathologists (CAP)-accredited KFSHRC core laboratories. In selected earlier cases with strong clinical suspicion, direct Sanger sequencing of candidate genes was undertaken. For this, genomic DNA was extracted from peripheral blood samples obtained via venipuncture, and Sanger sequencing was performed using BigDye Terminator chemistry (Thermo Fisher Scientific).

For in-house next-generation sequencing, libraries were prepared using standard Illumina protocols and sequenced on the NovaSeq platform. Sequence reads were aligned to the human reference genome, followed by variant calling and annotation as previously described (9). Identified variants were assessed against population databases, including the Saudi Human Genome Database, and evaluated for pathogenicity using established *in silico* prediction tools.

### 2.4 Preparative regimen, transplantation, and supportive care

All Patients received standardized antimicrobial prophylaxis from the time of diagnosis that included Trimethoprim-sulfamethoxazole for Pneumocystis jirovecii and antifungal prophylaxis with Fluconazole. Seven patients underwent HSCT at a median age of 4 (range, 2–8) months. High-resolution molecular human leukocyte antigens (HLA) typing was performed for class I (HLA-A, HLA-B, HLA-C) and class II (HLA-DRB1, HLA-DQB1) loci. Stem cell sources included unmanipulated bone marrow from HLA-matched sibling donors (n = 3), haploidentical parental donors (n = 2), and unrelated umbilical cord blood donors (n = 2). Patients who received transplants from HLA-matched related donors were conditioned using busulfan and cyclophosphamide, with or without anti-thymocyte globulin (ATG). Among the recipients of umbilical cord blood grafts, one received reduced-intensity conditioning consisting of fludarabine, melphalan, thiotepa, and ATG, whereas the others received a myeloablative regimen encompassing busulfan, cyclophosphamide, and ATG. The conditioning regimens for haploidentical transplantation included combinations of treosulfan or busulfan with fludarabine, thiotepa, and ATG. The dose of infused CD34^+^ cells ranged from 4.86 to 10.43 × 10^6^ cells/kg in transplants from HLA-matched or haploidentical donors. For umbilical cord blood transplants, the CD34^+^ cell dose ranged from 0.18 to 0.62 × 10^6^ cells/kg, with a range of total nucleated cell doses of 24.8 to 25.44 × 10^8^ cells/kg. Graft-versus-host disease (GVHD) prophylaxis consisted of cyclosporine and methotrexate for HLA-matched transplants, cyclosporine and corticosteroids for umbilical cord blood recipients, and posttransplant cyclophosphamide followed by cyclosporine and mycophenolate mofetil for haploidentical transplants.

All patients were managed in positive-pressure isolation rooms throughout the transplantation period. During the aplastic phase, patients received antimicrobial prophylaxis consisting of Pentamidine for Pneumocystis jirovecii, antiviral prophylaxis with Acyclovir, and antifungal prophylaxis with Voriconazole. Febrile episodes during aplasia were empirically treated with broad-spectrum intravenous antibiotics. Supportive care included transfusion of irradiated, cytomegalovirus (CMV)-negative red blood cells and platelets as clinically indicated. Preemptive antiviral prophylaxis with intravenous acyclovir (1500 mg/m²/day for 60 days) was administered to CMV-negative recipients with CMV-positive donors. G-CSF was administered at a dose of 5 μg/kg/day until the absolute neutrophil counts exceeded the limit of 0.5 × 10^9^/L. All patients received intravenous immunoglobulin replacement every 3 weeks.

### 2.5 Assessment of engraftment and GVHD

Donor chimerism was evaluated via short tandem repeat analysis. Acute and chronic GVHD were diagnosed based on established consensus criteria, with histopathological confirmation performed when indicated. Corticosteroids were used as the first-line treatment for GVHD, and additional immunomodulatory therapies were administered based on clinical severity and response.

## 3 Results

### 3.1 Patient characteristics and clinical presentation

**Figure 1:**
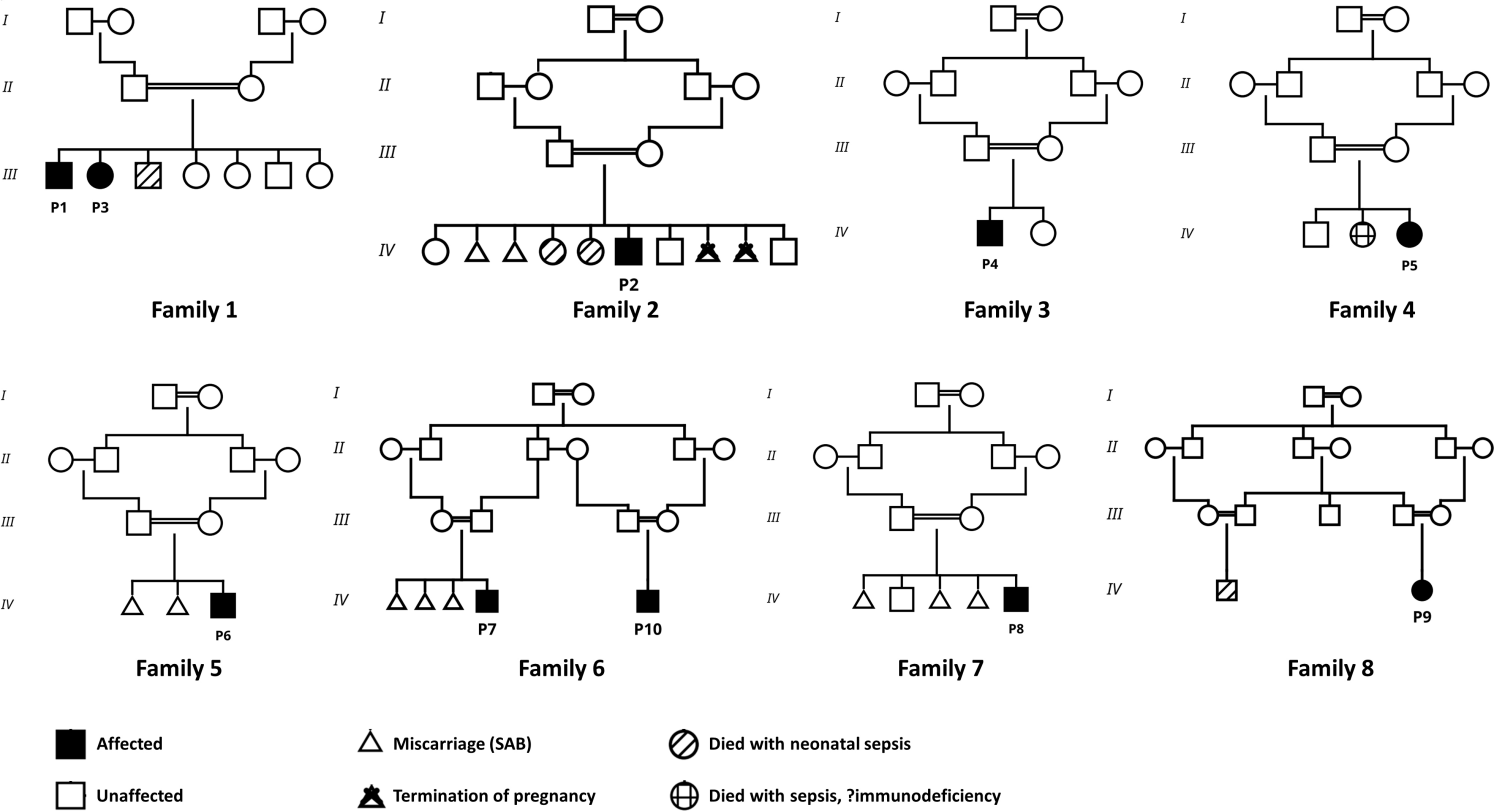
Pedigrees of eight unrelated families with AK2-associated disease. Filled symbols indicate affected individuals and open symbols indicate unaffected individuals. Across multiple families, recurrent patterns of affected offspring within sibships, often in the context of consanguineous unions, are observed. Pregnancy outcomes are annotated as follows: triangles denote miscarriage (spontaneous abortion, SAB), and crossed triangles denote termination of pregnancy. Individuals who died with neonatal sepsis (no genetic workup) are shown with diagonally striped symbols. One child who died with sepsis with suspected immunodeficiency is indicated by a crosshair-marked symbol. Generations are labelled with Roman numerals (I–IV).

A total of 10 patients from 8 unrelated families with genetically confirmed *AK2* deficiency were included in the analysis. Patient 1 (P1) has been previously described in a multicenter cohort study (7). Parental consanguinity was present in all patients. All family pedigrees are shown in **Figure 1**. The cohort consisted of seven males and three females. Six patients had a family history suggestive of primary immunodeficiency, whereas three had a family history of recurrent pregnancy loss. Two patients were diagnosed during targeted newborn screening based on a known familial history of immunodeficiency. Six patients were born prematurely. All patients presented with low birth weight, with three of them classified as very low birth weight (a median birth weight of 1.72 kg). The median age at initial clinical presentation was 14.4 days.

Neonatal sepsis was the most common presenting feature, occurring alongside profound neutropenia refractory to G-CSF therapy. Bilateral sensorineural hearing loss occurred in all patients. Pathogenic microorganisms were extracted from blood, wound, urine, or stool cultures. The pathogens identified were predominantly bacterial. Gram-positive organisms included *Staphylococcus epidermidis*, *Enterococcus faecalis*, *Staphylococcus aureus*, and *Bacillus licheniformis*, whereas Gram-negative isolates included *Aeromonas hydrophila*, *Enterobacter cloacae*, *Klebsiella pneumoniae*, *Escherichia coli*, and *Serratia marcescens*. The only viral pathogen detected was rotavirus, which was identified via gastrointestinal polymerase chain reaction test. A comprehensive summary of demographic data, clinical features, and infectious complications for each patient is presented in **Table 1**.

**Table 1:** Clinical features, and infectious complications pre-HSCT.

Patient	P1	P2	P3	P4	P5	P6	P7	P8	P9	P10
Sex	Male	Male	Female	Male	Female	Male	Male	Male	Female	Male
GA (weeks)	27	38	29	35	39	38	30	38	35	33
Birth weight (kg)	1.1	2.1	1.1	1.8	2.5	2.1	1.5	3.5	1.64	1.6
Weight percentile	< 3^rd^ percentile	< 3^rd^ percentile	< 3^rd^ percentile	< 3^rd^ percentile	5^th^ percentile	5^th^ percentile	< 3^rd^ percentile	50^th^ percentile	< 3^rd^ percentile	< 3^rd^ percentile
Onset of illness (days)	1	3	14	2	30	1	1	1	1	1
Clinical Presentation	Neonatal sepsis	Febrile neutropenia	Neonatal sepsis, NEC, thrombocytopenia	IUGR, Small ASD II, Cholestasis, bacteremia, NEC	Neonatal sepsis	Polyhydramnios, prolonged NICU admission with neutropenia.	Febrile neutropenia, otitis media, diarrhea, Cardiac: small stenotic collaterals with mild left chamber dilatation	Chronic diarrhea, skin rash, neutropenia and thrombocytopenia, sepsis	Erythroderma, petechial skin rash and pancytopenia Later on, developed diarrhea	IUGR, Severe neutropenia, RDS and small ASD
*Organisms isolated*	S. epidermidis	A. hydrophila, E. cloacae	MRSA	K. pneumoniae	E. coli, E. faecalis	E. coli, E. faecalis	S. aureus	E. coli, E. faecalis, MRSA	Rotavirus, S. marcescens	E. faecalis, B. licheniformis, A. fumigatus
Hearing loss	Yes	Yes	ND	Yes	Yes	Yes	Yes	Yes	Yes	Yes
Received HSCT	Yes	Yes	No	Yes	Yes	Yes	Yes	Yes	No	No

GA, gestational age; ASD II, secundum arterial septal defect; NEC, necrotizing enterocolitis; NICU, neonatal intensive care unit.

### 3.2 Laboratory and immunologic characteristics

Across the cohort, the median white blood cell count at presentation was 1.04 × 10^9^/L, with median absolute neutrophil and lymphocyte counts of 0.045 × 10^9^/L and 0.4 × 10^9^/L, respectively. Thrombocytopenia was reported in four patients. Five patients underwent bone marrow aspiration as part of the initial diagnostic evaluation, and all patients demonstrated a marked absence of granulopoiesis. The median lymphocyte subset counts revealed severe lymphopenia across all major lymphocyte populations, including CD3^+^ T cells (58.5 cells/mm³), CD4^+^ T cells (32 cells/mm³), CD8^+^ T cells (19 cells/mm³), CD19^+^ B cells (97.5 cells/mm³), and CD56^+^/CD16^+^ NK cells (42 cells/mm³). T-cell function was assessed in four patients via lymphocyte proliferation assays in response to PHA stimulation, showing severely impaired responses ranging from 0% to 3% of those observed in healthy controls. Serum IgG levels were within age-appropriate reference ranges at the time of evaluation, likely reflecting transplacentally acquired maternal immunoglobulin or continued intravenous immunoglobulin replacement therapy. all patients exhibited low IgA and IgM levels, that could be consistent with age-related physiological levels in early infancy. Detailed immunological findings at the time of initial presentation for each patient are summarized in **Table 2**.

**Table 2:** Genetic, laboratory and immunological characteristics at the time of initial presentation (pre-HSCT).

Patient	P1	P2	P3	P4	P5	P6	P7	P8	P9	P10	Reference ranges
*AK2 variant*	c.524G>C p.Arg175Pro	c.524G>C p.Arg175Pro	c.524G>C p.Arg175Pro	c.524G>C p.Arg175Pro	c.524G>C p.Arg175Pro	c.524G>C p.Arg175Pro	c.524G>C p.Arg175Pro	c.1A>G p.?	c.524G>C p.Arg175Pro	c.524G>C p.Arg175Pro	-
Zygosity	Homozygous	Homozygous	Homozygous	Homozygous	Homozygous	Homozygous	Homozygous	Homozygous	Homozygous	Homozygous	
SCID phenotype	T-B-NK-	T-B+NK+	T-B-NK-	T-B-NK+	T-B+NK+	T-B-NK+	T-B-NK-	T-B-NK-	T-B+NK-	T-B-NK-	-
*WBC (count 10^9^/L)*	0.63	1.03	1.05	1.89	0.51	1.61	1.42	2.25	0.45	0.32	4.30-11.30
*Neutrophils (count 10^9^/L)*	0.06	0.02	0.02	0.36	0.01	0.05	0.16	0.51	0.04	ND	1-6
*Lymphocytes (count 10^9^/L)*	0.45	0.84	0.36	0.07	0.44	1.27	0.6	0.27	0.034	ND	4-12
*Monocytes (count 10^9^/L)*	0.02	0.12	0.25	1.25	0.04	0.24	0.06	1.26	0.023	ND	0.03-1.00
*Hemoglobin (g/L)*	89	101	106	77	92	85	93	88	131	75	140-220
*Platelet (count 10^9^/L)*	349	392	24	35	83	956	862	617	17	234	150-450
*CD3+ (count/μL)*	99	35	451	158	16	1087	6	13	56	61	3100-4800
*CD3+/CD4+ (count/μL)*	74	20	226	95	16	44	3	7	18	58	2200-3300
*CD3+/CD8+(count/μL)*	23	15	241	51	0	1024	4	10	31	2	1100-1700
*CD19+(count/μL)*	98	248	5	11	617	97	85	433	187	0	1100-1900
*CD16+/CD56+(count/μL)*	12	160	5	360	83	404	33	27	51	3	300-700
*IgG (g/L)*	9.8 *	10 *	9 *	2	5 *	4 *	7.9 *	4.6	5.4	<3	2.5-9.1
*IgA (g/L)*	< 0.25	< 0.25	< 0.05	< 0.05	< 0.05	< 0.05	< 0.5	<0.5	0.1	<0.5	0.2-1.2
*IgM (g/L)*	< 0.18	< 0.18	0.18	< 0.3	0.30	< 0.3	< 0.25	<0.25	<0.25	<0.25	0.2-1.5
*Blastogenesis, PHA (CPM)*	(754) 0% of the control	(203) 0% of the control	ND	(2.895) 3% of control	(543) 0% of the control	ND	ND	ND	ND	ND	94935-171149
*BMA*	Hyperplastic erythropoiesis and megakaryopoiesis, almost absent granulopoiesis	Most of the cells are with immature phenotype. (suboptimal cell number)	ND	Myelopoiesis are reduced and arrested at promyelocyte stage with rare neutrophils.	No mature lymphocytes	Cellular marrow with active erythropoiesis and megakaryopoiesis and reduced granulopoiesis with complete absence of mature and maturing granulocyte	ND	ND	ND	ND	-

S epidermidis Staphylococcus epidermidis; E cloacae, Enterobacter cloacae; A hydrophila, Aeromonas hydrophila; MRSA, Methicillin-resistant Staphylococcus aureus; K pneumonia, Klebsiella pneumonia; E Coli, Escherichia coli; E faecalis, Enterococcus faecalis; S Aureus, Staphylococcus aureus; S marcescens, Serratia marcescens; B licheniformis, Bacillus licheniformis; A fumigatus; Aspergillus Fumigatus; WBC, White blood cell count; PHA, Phytohemagglutinin T cell activation; BMA, bone marrow aspiration; On IVIG replacement *ND, Not done. Normal references are provided for infants 1–4 weeks of age.

### 3.3 Molecular genetic testing

**Figure 2:**
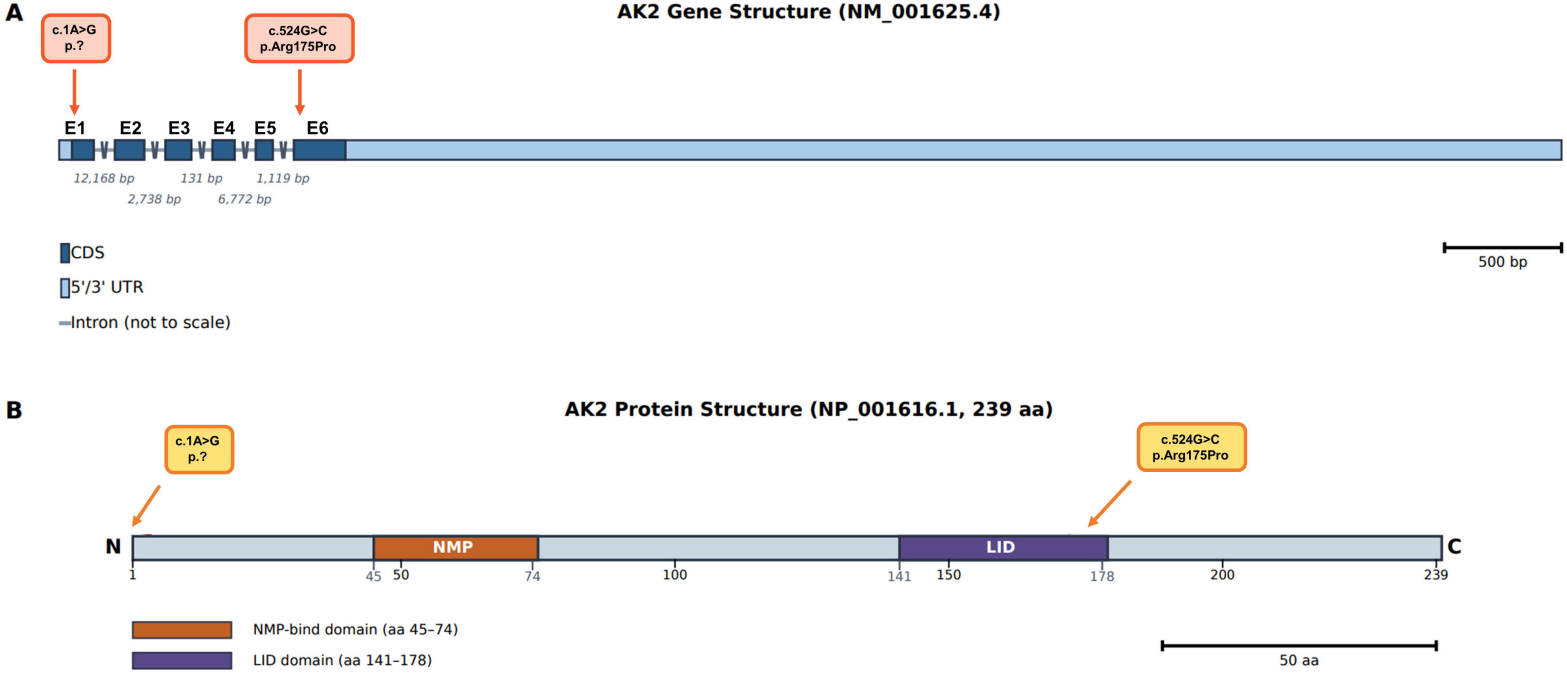
Schematic representation of the AK2 gene and protein structures with identified variants. **(A)** Genomic organization of AK2 (NM_001625.4), showing exons, coding regions and untranslated regions. Introns are not drawn to scale but their sizes are indicated. **(B)** Linear schematic of the AK2 protein (239 amino acids). The NMP-binding domain (aa 45–74) and LID domain (aa 141–178) are highlighted. The start-loss variant (c.1A>G; p.)? affects the initiating methionine at the N-terminus, while the p.Arg175Pro substitution localizes within the LID domain.

Molecular analysis revealed homozygous pathogenic variants in *AK2* in all 10 patients. Nine patients from nine unrelated families carried the same homozygous missense variant, NM_001625.4: c.524G>C (p. Arg175Pro) (**Figure 2A**), which has been previously documented in association with RD (7, 8). This variant causes an arginine-to-proline substitution at codon 175 of the AK2 protein (**Figure 2B**). Arginine 175 is a highly conserved amino acid located within the catalytic core of AK2, a region crucial for nucleotide binding and phosphotransfer activity. Replacement of arginine, a positively charged and conformationally flexible residue, with proline, a structurally rigid amino acid known to disrupt secondary protein structure, is predicted to cause substantial conformational instability and loss of enzymatic function.

One patient (P8) harbored a previously reported homozygous variant affecting the translation initiation codon, NM_001625.4: c.1A>G (p.)? (**Figures 2A, B**). In the absence of an alternative functional downstream start site, this start-loss variant is expected to cause complete loss of AK2 protein expression, consistent with a loss-of-function mechanism (6).

### 3.4 HSCT engraftment, survival, and toxicity

Three patients did not undergo HSCT. One patient died after rapid clinical deterioration due to an invasive fungal infection, whereas the other two patients succumbed to sepsis prior to the planned transplant. Seven patients underwent HSCT. The overall posttransplant survival rate was 85.71% (6/7) after a median follow-up of 10 years (range, 1–18). One patient (P7) died after haploidentical transplantation due to transplant-related complications, including septicemia.

All transplanted patients achieved full donor engraftment, with 100% donor chimerism in myeloid and lymphoid lineages. The median time to neutrophil recovery, defined as an absolute neutrophil count greater than 0.5 × 10^9^/L, was 17 days. Platelet recovery, characterized by a platelet count > 20 × 10^9^/L without transfusion support, occurred at a median of 32.5 days. One patient developed acute GVHD of the skin, which responded to systemic corticosteroid therapy. There were no cases of chronic GVHD. Infectious complications following transplantation included skin infection and septicemia in five patients. Isolated organisms included *E. coli*, *K. pneumoniae*, and *Aeromonas hydrophilia*.

After HSCT, all the surviving patients exhibited marked improvement in hematologic and immunologic parameters, including normalization of white blood cell, neutrophil, and lymphocyte counts. The median posttransplant lymphocyte subset counts were as follows: CD3^+^ T cells, 2760.5 cells/mm³; CD4^+^ T cells, 1636.5 cells/mm³; CD8^+^ T cells, 918.5 cells/mm³; CD19^+^ B cells, 1060 cells/mm³; and CD56^+^/CD16^+^ NK cells, 330.5 cells/mm³. Patients who underwent functional testing showed improved lymphocyte proliferative responses to PHA stimulation, (90%–106% of the control).

Intravenous immunoglobulin replacement therapy was discontinued in five of the six surviving patients, all of whom subsequently achieved protective antibody responses following routine immunization. Patient 8 is currently 1-year post-haploidentical HSCT with successful B-cell engraftment, and discontinuation of immunoglobulin replacement therapy is planned over the next few months. Detailed transplant outcomes and immune reconstitution data at last follow up are presented in **Table 3**.

**Table 3:** Hematopoietic stem cell transplantation and immune reconstitution at last follow up.

Patient	P1	P2	P4	P5	P6	P7	P8	Reference range
*Age at HSCT*	7 months	2 months	4 months	3 months	5 months	3 months	8 months	-
*year of hsct*	2008	2011	2014	2017	2017	2022	2025	
*Stem cells source*	Sister	Umbilical cord	Umbilical cord	Brother	Brother	Father	Father	-
*HLA match*	10/10	6/6	5/6	10/10	10/10	5/10	5/10	–
*Conditioning regimen*	BU/Cy	BU/Cy/ATG	FLU/MEL/ TESPA/ATG	BU/FLU	BU/FLU/ATG	BU/FLU/ATG/TESPA	TESPA/TREO/FLU/ATG	-
*GVHD prophylaxis*	CSA/MTX	CSA/MTX	CSA/Steroid	CSA/MTX	CSA/MMF/MTX	CSA/PT-CY/MMF	PT-CY/CSA,MMF	–
*CD34 dose/kg (10^6/^kg)*	8.47	0.18	0.62	8.12	10.43	5.44	4.86	-
*Complications*	Septicemia	Skin infection	Septicemia	Septicemia	Septicemia	Multiorgan failure, septic shock, suspected VOD	None	
*Acute GVHD*	Skin	-	-	-	-	Gut (GI bleed)	-	-
*Chronic GVHD*	–	–	–	–	–	–	–	–
*Neutrophil engraftment (days)*	+16	+24	+17	+20	+18	15+	14+	-
*Platelet engraftment (days)*	+23	+51	+31	+35	+34	Did not engraft	23+	–
*Lymphoid donor chimerism (%)*	100	100	100	100	100	100	100	-
*Myeloid donor chimerism (%)*	100	100	100	100	100	100	100	–
*WBC*	8.16	6.37	7.11	6.95	8.67	4.13	5.92	4.30-11.30
*Neutrophil count*	1.83	1.26	1.77	1.28	1.64	3.01	2.86	1-6
*Lymphocyte count*	4.99	4.51	3.56	4.03	5.29	ND	1.60	4-12
*CD3+ (count/μL)*	3242	2587	2485	2944	4235	ND	1865	2700 - 6400
*CD3+ CD4+ (count/μL)*	1423	1798	1718	1555	2932	ND	1418	1025 - 3498
*CD3+ CD8+ (count/μL)*	1887	702	560	1161	1135	ND	269	132 - 1530
*CD19+ (count/μL)*	547	1368	2252	1505	752	ND	399	131 - 1271
*CD16+/CD56+ (count/μL)*	595	282	785	151	295	ND	366	536 - 2989
*Naive T cell CD4,CD45RA (count/μL)*	36	26	17	ND	40	ND	ND	354 - 1679
*Blastogenesis, PHA (CPM)*	117,973 (90% of control)	246,894 (134% of control)	160,453 (106% of control)	ND	ND	ND	ND	94,935-171,149
*IgA (g/L)*	0.90	0.43	0.68	1.15	3.07	ND	0.61*	0.05 – 0.4
*IgM (g/L)*	1.11	0.81	0.8	0.97	1.35	ND	0.68*	0.15 – 0.7
*IgG (g/L)*	8.7	6	6.1	7	14.1	ND	18.7*	2.1 – 7.7
*IVIG*	No	No	No	No	No	ND	Yes	–
*Outcome*	Alive, vaccinated. Appropriate growth. Required hearing aids.	Alive and vaccinated. Appropriate growth. Underwent cochlear implantation. Hypothyroidism on treatment.	Alive and vaccinated. Underwent cochlear implantation. Appropriate weight gain but with short stature.	Alive and vaccinated. Poor weight gain, normal height. Underwent cochlear implantation.	Alive, normal immune reconstruction. Frequent admissions with nephrogenic DI and bronchiectasis Having FTT and short stature, post cochlear implant	Death post transplant due to sepsis	Alive, yet to receive vaccination	-
*Follow up (years)*	18	15	12	9	9	–	1	–

HLA, Human Leukocyte Antigen; Cy, Cyclophosphamide; ATG, anti-thymocyte globulins; FLU, fludarabine; MEL, melphalan; BU, Busulfan; TESPA, Theotipa; TREO, Treosulphan; CSA, cyclosporine; MTX, Methotoxate; MMF, mycophenolate mofetil; PT-Cy, Post-transplant Cyclophosphamide; ND, not done; PHA, Phytohemagglutinin T cell activation; IVIG, intravenous immunoglobulin; GVHD, graft versus host disease; VOD, veno-occlusive disease. Normal reference of lymphocyte subset ranges provided for infants 4 to 12 weeks old. Immunoglobulin normal ranges provided for infants 6 to 12 weeks of age.

### 3.5 Hearing defect and development

All patients had bilateral sensorineural hearing loss. Of them, four underwent cochlear implantation following HSCT, whereas one required hearing aids. One patient was diagnosed with hypothyroidism and commenced appropriate replacement therapy. Two patients exhibited short stature and poor weight gain.

## 4 Discussion

Reticular dysgenesis (RD) is traditionally classified within Severe Combined Immunodeficiency due to its profound T-cell deficiency and early-onset clinical presentation. However, RD is biologically distinct from classical SCID, as it is characterized by severe congenital neutropenia and a primary defect at the level of hematopoietic stem and progenitor cells driven by mitochondrial dysfunction. Unlike typical SCID, which predominantly affects lymphoid development, RD reflects a broader failure of hematopoiesis involving both lymphoid and myeloid lineages. This dual-lineage impairment has important clinical and therapeutic implications, including increased susceptibility to bacterial and fungal infections and potential challenges in achieving sustained engraftment following hematopoietic stem cell transplantation. Accordingly, RD may be more appropriately conceptualized as a disorder at the intersection of SCID and inherited bone marrow failure syndromes. It represents a distinct entity within the spectrum of inborn errors of immunity, characterized by combined lymphoid and myeloid failure, with implications for classification, management, and future research strategies.

The clinical presentation of patients in our cohort closely aligns with previously reported cases of RD, suggesting a distinct phenotype within the spectrum of SCID. All patients presented early in life, predominantly within the neonatal period, with severe bacterial infections and neutropenia refractory to G-CSF. This pattern reflects findings from the international multicenter survey, in which >90% of patients presented within the first month of life, most commonly with bacterial sepsis rather than opportunistic infections (7). Similarly, the universal presence of bilateral sensorineural hearing loss in our cohort highlights its role as a hallmark clinical feature of RD and a critical diagnostic indicator distinguishing RD from other SCID entities. The predominance of bacterial pathogens and the relative absence of opportunistic infections further indicate that agranulocytosis, rather than isolated lymphocyte dysfunction, is the dominant driver of early morbidity in RD.

A notable finding in our cohort is the high prevalence of prematurity and low birth weight, with 60% of patients born preterm and all patients exhibiting low birth weight, including several with very low birth weight. These findings closely align with previous reports indicating increased frequency of prematurity and small-for-gestational-age status among patients with RD. Although the precise mechanisms remain unclear, it has been hypothesized that AK2 plays a pivotal role in fetal cellular energy homeostasis, and its deficiency may disrupt intrauterine growth and hematopoietic development (4). Previous studies have indicated that growth impairment is not progressive after successful HSCT, suggesting that postnatal growth and development are not intrinsically limited by the genetic defect itself. Our findings further indicate that prematurity and growth restriction are part of the disease phenotype rather than consequences of postnatal illness alone.

All patients harbored homozygous pathogenic variants in *AK2*, reflecting the high consanguinity rate observed in our cohort. The recurrence of an identical homozygous variant across unrelated families suggests a possible founder effect, although this requires confirmation through population-based genetic studies. Despite the increasing recognition of diverse *AK2* pathogenic variants, including missense, nonsense, and splice-site variants, a consistent genotype–phenotype correlation has not yet been established. This likely reflects the fact that different variants can lead to a similar functional outcome, namely impaired AK2 activity. Experimental models support this concept, demonstrating that both partial and complete loss of AK2 function disrupt mitochondrial energy homeostasis, increase oxidative stress, and compromise the survival and differentiation of hematopoietic stem and progenitor cells (4).

HSCT remains the only curative therapy for RD, and the outcomes in our cohort favorably align with those reported in the literature. We observed an overall posttransplant survival rate of 85.71%, exceeding the approximately 68% reported in the largest international series (7). When contextualized within the broader field of severe combined immunodeficiency, these outcomes are comparable to survival rates reported for other SCID subtypes, where overall survival typically ranges between 70% and 90% depending on donor type, conditioning strategy, and timing of transplantation (1, 10–12). All transplanted patients in our study achieved full donor myeloid and lymphoid engraftment, indicating the effectiveness of conditioning-based transplant approaches. Previous studies have reported that myeloablative conditioning is crucial for achieving durable myeloid engraftment and preventing recurrent agranulocytosis, which is a key determinant of long-term survival in RD (4, 7). Mixed chimerism in reticular dysgenesis may permit partial immune reconstitution, particularly when donor T-cell engraftment is achieved; however, sustained disease correction depends on adequate donor-derived myeloid engraftment due to the intrinsic stem cell defect. Incomplete myeloid engraftment carries several important risks. These include persistent or recurrent neutropenia, ongoing susceptibility to bacterial and fungal infections, and the potential need for additional interventions such as growth factor support or second transplantation. Furthermore, inadequate myeloid reconstitution may compromise long-term hematopoietic stability, even in the presence of satisfactory lymphoid recovery. Together, these considerations underscore the importance of achieving robust donor myeloid engraftment to ensure sustained disease correction and optimal clinical outcomes. The low incidence of GVHD, observed in our cohort, further supports the feasibility and safety of HSCT in this vulnerable population. This favorable GVHD profile likely reflects careful donor selection, optimized conditioning strategies, and effective supportive care. Importantly, immune reconstitution following HSCT was robust, with recovery of both cellular and humoral immunity. This enabled the discontinuation of immunoglobulin replacement therapy and the development of protective responses to routine vaccinations in surviving patients, indicating successful long-term immune competence.

Beyond allogeneic HSCT, personalized gene therapy represents a logical future direction for RD, particularly in the context of autologous transplantation. As the disease is driven by loss-of-function variants in *AK2* affecting hematopoietic stem and progenitor cells, *ex vivo* gene correction of patient-derived CD34+ cells followed by autologous reinfusion could, in principle, restore myelopoiesis while avoiding GVHD and donor limitations. Recent work using CRISPR/Cas9 gene editing with adeno-associated viral vector delivery in human HSPCs has provided mechanistic insight into metabolic checkpoint failure in RD while also illustrating the feasibility of contemporary gene correction strategies (13). Key challenges remain however, including achieving stable gene correction in long-term repopulating stem cells, as well as ensuring durable myeloid engraftment. While clinical application is not yet established, advances in gene therapy for other primary immunodeficiencies support continued translational development in RD.

This study provides several important contributions that extend beyond previously published reports on *AK2*-related reticular dysgenesis. First, it represents the largest single-center cohort reported to date, with detailed and longitudinal follow-up extending up to 18 years, allowing for a comprehensive assessment of long-term transplantation outcomes and immune reconstitution. Second, the study offers a uniquely consistent clinical and immunologic characterization of patients managed within a single institution, reducing variability in diagnostic and therapeutic approaches that often limits multicenter analyses. Importantly, it demonstrates favorable survival and robust immune reconstitution following hematopoietic stem cell transplantation across different donor sources, contributing practical evidence to guide clinical decision-making. However, it has several limitations that need to be acknowledged. First, the retrospective design and relatively small sample size reflect the extreme rarity of RD and limit the ability to conduct formal statistical comparisons or genotype–phenotype analyses. Second, transplant strategies changed over the long study period, resulting in heterogeneity in conditioning regimens and donor selection. Third, functional immune testing and long-term endocrine and neurodevelopmental follow-up were not consistently available for all patients. Addressing these limitations provides a clear framework for future research. Future studies should prioritize prospective, multicenter collaboration to allow standardized data collection and more robust comparative analyses. Such studies would enable systematic evaluation of conditioning intensity, donor selection, and graft source, addressing the heterogeneity of transplant strategies observed across cohorts. Furthermore, a long-term, standardized follow-up is crucial to better characterize immune reconstitution, growth patterns, endocrine and neurodevelopmental outcomes, and quality of life in survivors. In addition, mechanistic investigations into mitochondrial dysfunction and oxidative stress in *AK2* deficiency may facilitate the identification of adjunctive therapeutic strategies.

Saudi Arabia has one of the highest reported incidences of SCID worldwide, driven largely by consanguinity and large family size, resulting in recurrent familial cases and early neonatal mortality (14). The serious neonatal presentation, high pretransplant mortality, and rapid clinical deterioration observed in our cohort further underscore the limitations of symptom-based diagnosis. These observations strongly support the implementation of population-based newborn screening for SCID, which would enable presymptomatic diagnosis, early infection prevention, and timely referral for curative HSCT. Considering the survival advantage associated with early transplantation, integrating SCID screening into national newborn screening programs is a critical public health priority in Saudi Arabia and similar high consanguinity populations.

## 5 Conclusion

*AK2*-related RD is a severe SCID variant characterized by early neonatal presentation, agranulocytosis, and sensorineural hearing loss, carrying high mortality if not treated promptly. Our single-center experience demonstrates that early HSCT can achieve durable engraftment, immune reconstitution, and favorable survival. The high consanguinity rate and SCID burden in Saudi Arabia emphasize the limitations of symptom-based diagnosis and highlight the urgent need for population-based newborn screening for SCID to allow early intervention and enhance outcomes.

## Data Availability

The dataset used in this study is protected under privacy law and cannot be publicly available. Requests for access to the data may be directed to the corresponding author at: hamoudalmousa@kfshrc.edu.sa.
